# Views on high-fat diet linked insulin resistance

**DOI:** 10.6026/97320630018050

**Published:** 2022-01-31

**Authors:** Priscilla Daniel, Madhan Krishnan, Monisha Prasad, Shyamaladevi Babu, TM Vijayalakshmi

**Affiliations:** 1 Department of Medical Biochemistry, Dr. A.L.M PG Institute of Basic Medical Sciences, University of Madras, Chennai, Tamil Nadu, India; 2 Department of Biochemistry, Vels Medical college & Hospital, Vels Institute of Science Technology & Advance Studies (VISTAS), Chennai, Tamilnadu, India; 3 Department of Biochemistry, Saveetha Institute of Medical and Technical Sciences, Chennai, Tamilnadu, India

**Keywords:** Diabetes, high-fat diet, insulin resistance

## Abstract

Insulin resistance is linked to impaired cell metabolism and survival in the peripheral tissues, as well as increased oxidative stress and activated inflammatory responses. Chronic High fat diet insulin resistant to exposure results in liver damage, impaired glucose homeostasis, hyperinsulinemia, late pancreatic-cell failure to generate insulin due to cell exhaustion, and subsequent hyperglycaemia, all of which are hallmarks of Type 2 Diabetes Mellitus (T2DM). Therefore, it is of intrest to document a short review on the impact of a high-fat diet with insulin resistance.

## High fat diet to insulin resistance:

Impaired Triglycerides TG deposition and increased lipolysis result in an excess of circulating TG and FFAs Free fatty acids, which contributes to ectopic lipid accumulation in the liver and skeletal muscle, resulting in hepatic steatosis and insulin resistance [1-2]. CLDs (chronic liver diseases) are hypothesised to offer substrate for CM synthesis and other metabolic destinations such as complex lipid synthesis, oxidation, and signalling [3]. Insulin signalling leads to type 2 diabetes mellitus and obesity (the process of dietary fat absorption is changed) is common [4-5]. Obesity modifies peripheral tissues in response to insulin, where white adipose tissue, liver, and muscle playing major roles in the development of insulin resistance [6-7].

It is also known that the composition of FA (Fatty acid) intake could influence the characteristics of cell membranes and their FA components. Changes in the FA composition of the sarcolemma could modify membrane fluidity and stiffness [8]. The efficiency of the signal transduction is strongly dependent on the orientation and position of many proteins and the FA composition in the transduction of the insulin signal [9]. Transcription of genes involved in gluconeogenic control, including PCK1 and G6PC controled by insulin results in hepatic glucose production (HGP) [10].

Hyperglycemia and hyperinsulinemia has a significant effect on gluconeogenic genes, such as Pck1 and G6pc by double triumph of the insulin receptor substrates (IRS) namely, Irs1 and Irs2 in liver [11]. The activation of IRS proteins results in the recruitment of the lipid kinase PI3K to the plasma membrane, where it phosphorylates PI-(4,5)-bisphosphate (PtdIns(4,5)P2/PIP2) to generate PtdIns(3,4,5)P3 (PIP3), an important second messenger of several growth factor receptors and mediators of PEPCK and G-6-Pase expression levels along with the insulin-stimulated PI3K-mediated phosphorylation of Akt at Ser473 activates the kinase [12].

Insulin signalling via phosphorylation of FOXO1 by AKT contributes to the reported decrease in steady-state mRNA levels for gluconeogenic genes such G6pc and Pck1. FOXO1 causes the derepression and/or activation of the GLUT4 gene, which leads to improved insulin sensitivity [13]. A fundamental error in insulin signalling will invariably result in aberrant post-prandial lipid metabolism. Another possibility is that insulin resistance is caused by improper post-prandial lipid metabolism [14].

## Conclusion:

It is interest to document the effect of fat metabolism linked with insulin. Insulin resistance, is caused by lipid metabolic abnormalities and elevated levels of circulating fatty acids that accumulate in insulin-sensitive organs such muscle, liver, and adipose tissues. Severe diseases such as diabetes, obesity, stroke, and heart attack are developed, as a result of high fat diet, where insulin resistance is a risk factor that can be managed with therapeutic lifestyle changes and pharmacological therapy.
